# Aquatic Plant Mediates Microplastic Bioavailability in Herbivorous Freshwater Fish

**DOI:** 10.1007/s00244-025-01164-3

**Published:** 2025-10-29

**Authors:** Shinnosuke Yamahara, Yoichi Era, Haruhiko Nakata

**Affiliations:** 1 Graduate School of Science and Technology, Kumamoto University, 2-39-1 Kurokami, Chuo-ku, Kumamoto 860-8555 Japan; 2 Faculty of Advanced Science and Technology, Kumamoto University, 2-39-1 Kurokami, Chuo-ku, Kumamoto 860-8555 Japan

## Abstract

This study analyzed microplastics (MPs; target size: 0.1–5 mm) in freshwater fish and aquatic plants in an urban lake in Kumamoto, Japan. MPs were detected in 82% of the collected sample of herbivorous fish species, Nile tilapia (*Oreochromis niloticus*) and Redbelly tilapia (*Tilapia zillii*) at mean level of 10 ± 13 items ind^−1^, which was more than 10 times higher than the levels found in two carnivorous fish species, largemouth bass (*Micropterus salmoides*) and snakehead (*Channa argus*). Fish samples near the outlets of stormwater runoff and a wastewater treatment plant showed higher abundances of MPs than fish collected from other locations in the lake. Polyethylene (PE), polypropylene (PP), and polyethylene terephthalate (PET) were dominant polymers in fish, accounting for 83%. These three polymers were also commonly detected in three species of aquatic plants, which retained MPs at 4.6–35 items g^−1^ dry weight. A comparison of the characteristics of the MPs detected in the herbivorous fish and aquatic plant samples collected at the same station showed similar profiles in terms of polymer types, shape, and colors. This indicates that herbivorous fish are exposed to MPs adsorbed onto the surface of aquatic plants through their feeding activity. Our findings highlighted that aquatic plants act as a mediator to increase the bioavailability of MPs in freshwater fish by trapping MPs on the plant surface.

**Graphical Abstract:**

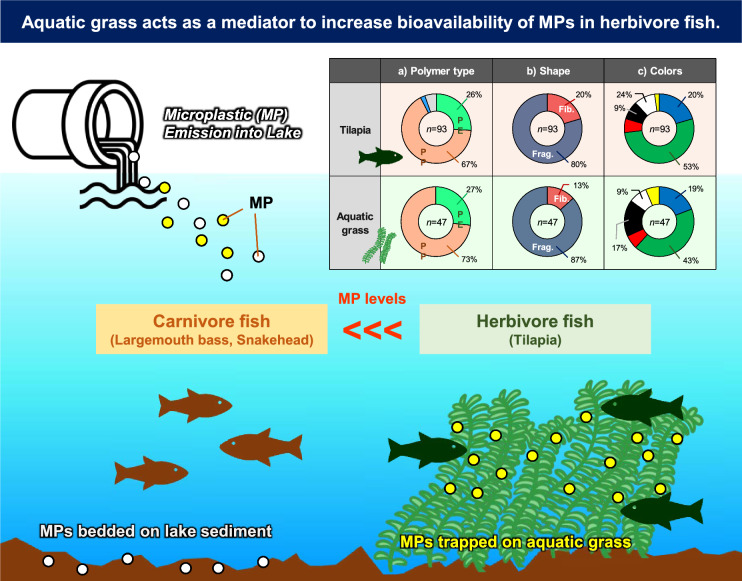

**Supplementary Information:**

The online version contains supplementary material available at 10.1007/s00244-025-01164-3.

## Introduction

Microplastics (MPs; plastic fragments < 5 mm; GESAMP 2016) pose an increasing pollution threat to aquatic environment. The ecological risks of MPs in organisms have been discussed from aspects of particle toxicity (Sussarellu et al. 2019), chemical toxicity derived from plastic additives (Tanaka et al. 2020) and adsorbed substances such as persistent organic pollutants (Ogata et al. 2009). Freshwater bodies are the area of high concern for potentially severe MPs pollution caused by extensive human activities (Yamahara and Nakata 2025).

Biomonitoring is an important approach to understand the MPs pollution in biota, the environmental distribution, and fate. Fish have been frequently investigated for MPs because of their commercial values and suitability as bioindicators of MPs pollution (Tanaka and Takada 2016; Bessa et al. 2018; Mejjad et al. 2024). A number of studies have discussed exposure pathways of fish species to MPs. For instance, Okamoto et al. (2022) proposed color recognition and preference of MPs by freshwater and marine fish can be associated with those exposures. Another study reported the importance of unintentional exposure to MPs in fish through ingesting water for osmoregulation (Pratiwi et al. 2023). In addition, the trophic transfer of MPs from small shrimp-like filter-feeder crustaceans (mysids for genus *Neomysis*) to a carnivorous fish (the sculpin, *Myoxocephalus brandti*) greatly exceeds the amount directly ingested by the fish from the water column (Hasegawa and Nakaoka 2021). After the exposure, MPs are retained in the guts and gills of fish and circulated and spread to other tissues, which influence a number of hematological parameters that are connected with immunity, osmotic pressure, blood clotting, molecular transport, and fat metabolism (Ghosh 2025). However, few studies focused on the exposure pathway of MPs in herbivorous fish even though they are reported to show higher level of MPs than carnivorous fish (Ahmed et al. 2023; Bhatt et al. 2024).

As the main diets of herbivorous fish, aquatic plants have been recently recognized as a sink of MPs in the aquatic environment (Huang et al. 2023). Previous studies have reported on the adverse effects of MPs on aquatic plant (Osman et al. 2023), the function of vegetated areas as a sink of MPs accumulation (Ng et al. 2022), and differences in MPs trapping ability by plant morphology (Ng et al. 2022). This indicates that fish may ingest different amounts of microplastics depending on their diet, with herbivorous fish in particular likely to have higher concentrations. However, information is still lacking about the presence of MPs on aquatic plant in relation to MPs exposure in herbivorous fish. We hypothesized that aquatic plant acts as a mediator to increase the bioavailability of MPs in herbivorous freshwater fish. Therefore, this study aimed to (i) examine the exposure levels of MPs in herbivorous freshwater fish and aquatic plants in an urban lake environment; (ii) compare the exposure levels of MPs between herbivorous and carnivorous fish species, and (iii) clarify the potential role of aquatic plant as a pathway of MPs exposure in freshwater fish.

## Materials and Method

### Study Area and Sampling

**Fig. 1:**
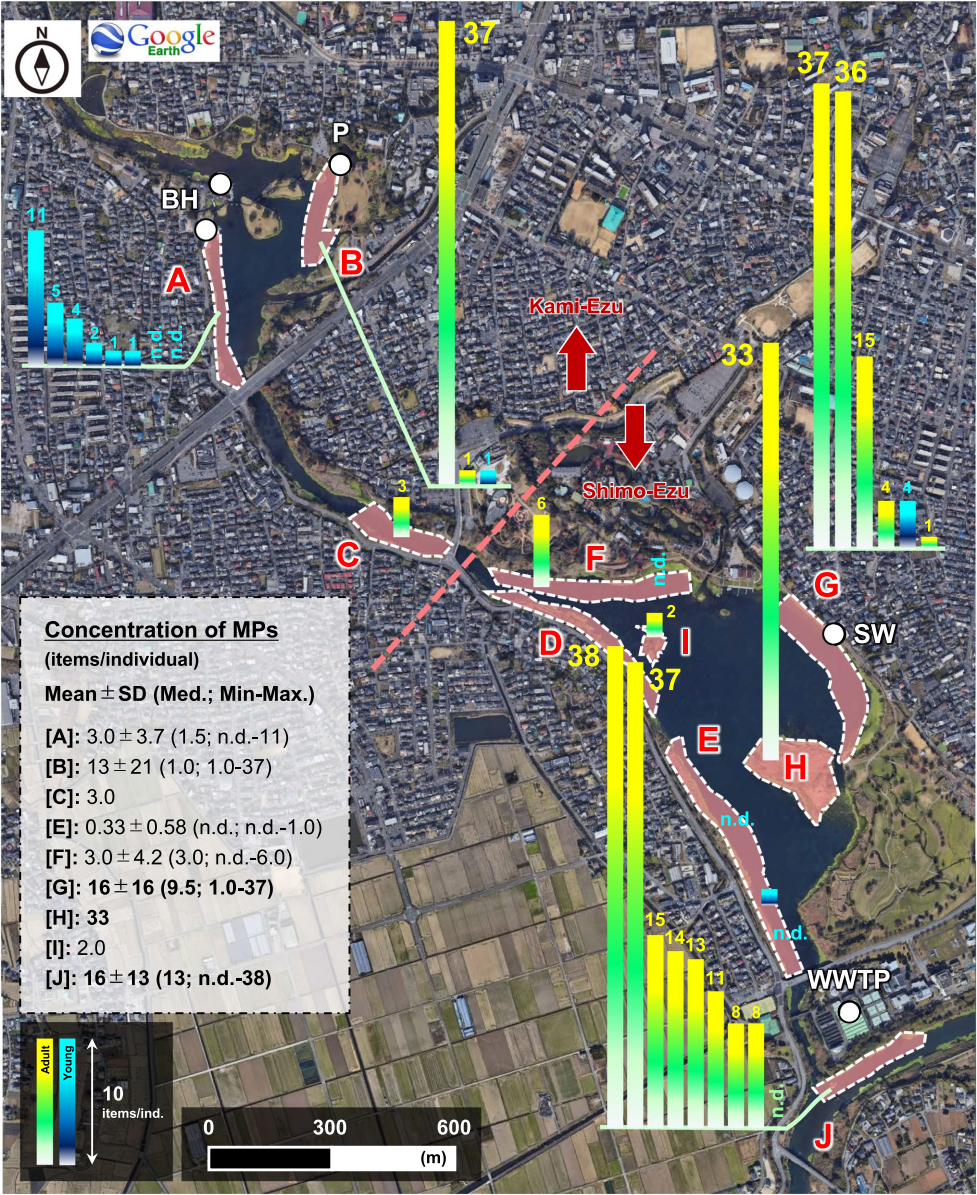
Spatial distribution of MPs abundances in stomach contents of tilapias. Each bar indicates individual specimen. Yellow: adult specimens (body size: > 18.6 cm), Blue: Juvenile specimens (body size: < 18.6 cm), BH: boat house, P: park where people are frequently visiting for recreation, SW: stormwater outlet, WWTP: wastewater treatment plant

This study was conducted in Lake Ezu, a small lake of approximately 50 ha and 1.2–2.0 m depth on average located in an urban area of Kumamoto City, Japan (https://www.ezuko-park.com/). Lake Ezu is actually made up of two small lakes, Kami-Ezu and Shimo-Ezu (Fig. 1). Sampling area was segmented into 10 stations (St. A to J; Fig. 1) from Kami-Ezu (St. A, B) and Shimo-Ezu (St. D–I). Boat house is located nearby St. A whose sediment has been reported to be highly contaminated with MPs derived from boat paint fragments (Era and Nakata 2020). The north part of St. B is a place, where ordinary citizens are frequently visiting. St. G and J are located nearby the outlet of stormwater runoff and WWTP, respectively.

During 2019 and 2021, three species of the freshwater fish were collected by a method of electrofishing from the lake: the mainly herbivorous tilapia (Nile tilapia [*Oreochromis niloticus*] and redbelly tilapia [*Tilapia zillii*]; *n* = 34), the carnivorous largemouth bass (*Micropterus salmoides*; *n* = 29), and snakehead (*Channa argus*; *n* = 22) (Fig. S1; Table S1). Matured Nile tilapia (size: > 18.6 cm) is mostly an herbivore fish, whereas it’s juveniles tend to be more omnivorous than adults (Froese 2024a). Largemouth bass and snakehead are carnivore species (Froese 2024b, c). All fish samples were immediately transported to the laboratory, and the stomach content was placed on clean aluminum foil. The stomach contents were stored at −20 ℃ until analysis. In addition, samples of three types of aquatic plants (*n* = 8) also collected in the lake: Brazilian waterweed (*Egeria densa*; *n* = 3), eelgrass (*Vallisneria higoensis*; *n* = 3), and large holly-leaf waternymph (*Najas marina*; *n* = 2) in 2020–2021 (Fig. S1). Some plants are dominant species in this lake (Shimizu 2017), and they are considered as a diet of herbivorous fish. A root part of aquatic plant was removed to avoid the contamination of MPs derived from lake sediment. All aquatic plant was dried at 60 ℃ and used for MP analysis.

### MP Analysis

The MP analysis followed the procedure described in our previous study (Yamahara et al. 2024a), with a slight modification. The whole stomach content of each fish and 1 g of dried and homogenized each aquatic plant were used for MP analysis. All samples were first treated with 100–200 mL of 30% hydrogen peroxide (H_2_O_2_; Fujifilm-Wako Pure Chemical Industries, Japan) at 50 ℃ for 3 days. The solid residue was density-separated with 60% sodium iodide solution (1.8 g/cm^3^; Fujifilm-Wako Pure Chemical Industries, Japan). A floating fraction (< 1.8 g/cm^3^) was transferred onto the 100 μm-mesh nylon filter, and this protocol was repeated three times. The residues on nylon filters were dried at 50 ℃ overnight, and each candidate of MPs was collected using tweezers under a stereomicroscope (S9, LEICA, Germany). Finally, the polymers of particles were determined using attenuated total reflection Fourier-transform infrared spectrometer (ATR-FT-IR; IR Affinity 1S, Shimadzu, Japan). The spectrum of each sample was compiled from 20 scans recorded over 600 to 4000 cm^−1^ at a resolution of 4 cm^−1^ with Happ-Genzel apodization. Background spectra were acquired every 3–5 samples, and the stage and crystal of FTIR were kept clean by wiping with isopropyl alcohol (Fujifilm-Wako Pure Chemical Industries, Japan) before each measurement. The threshold for polymer determination was a > 75% match to a reference spectrum in various libraries including the Aldrich Standard. The size of MPs was measured using a software of ‘Image J’ in this study.

### Quality Assurance and Quality Control (QA/QC)

To avoid contamination of the MPs, non-plastic clothing was worn during chemical analysis. In addition, all glass wares were carefully washed by ultrapure water and clean solvent (acetone and *n*-hexane) and covered with pre-cleaned aluminum foils during experiment to avoid dust contamination. In addition, a procedural blank sample was run every four samples. Several cellulose fibers were identified, but there was no contamination of MPs in all blank samples.

Moreover, spike and recovery tests (*n* = 9) were performed using handmade microplastics (250–1000 μm) and standard sand. Ten particles in each of polyethylene (PE), polyethylene terephthalate (PET), polyvinyl chloride (PVC), and polymethyl methacrylate (PMMA) were added into 5 g of standard sand and analyzed following the entire analytical procedure described above. The recoveries of PE, PET, PVC, and PMMA were 99 ± 9.5, 87 ± 13, 102 ± 3.4, 100 ± 0%, respectively.

### Statistical Analysis

The statistical analysis (Mann–Whitney *U* test) was performed using a software of Excel Statistics (Esumi Co Ltd, Tokyo, Japan).

## Results and Discussion

### Abundance and Spatial Distribution of MPs in Freshwater Fish

**Fig. 2:**
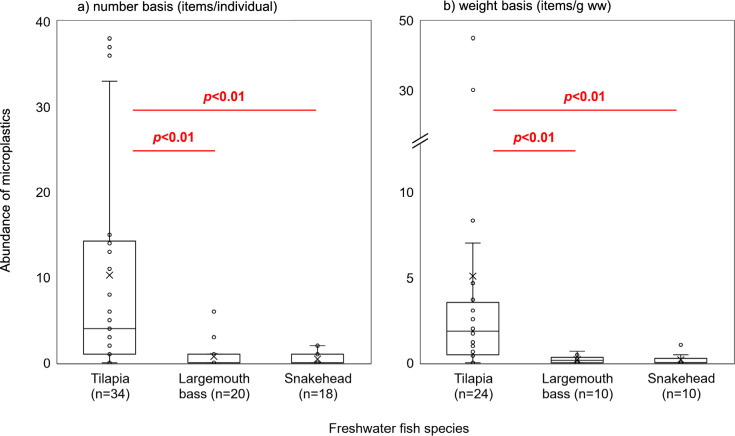
Comparison of microplastic abundance among freshwater fish. **a**: Number basis. **b**: Weight basis. *p* values were determined by Mann–Whitney *U*-test

A total of 372 MPs were identified in the stomach contents of fish samples. The herbivorous fish, tilapia, showed a significantly higher abundance of MPs (detection frequency [DF]: 82%, mean: 10 ± 13 items individuals^−1^ [items ind.^−1^]), 5.1 items g^−1^ of stomach content on a wet weight basis [g^−1^ ww]) than the carnivorous species, largemouth bass (DF: 40%, mean: 0.76 ± 1.4 items ind.^−1^, 0.19 ± 0.23 items g^−1^ ww) and snakehead (DF: 33%, mean: 0.44 ± 0.70 items ind.^−1^, 0.17 ± 0.36 items g^1^ ww) (Table 1; Fig. 2). The high-level exposure to MPs in herbivorous fish has been also reported in other recent studies (Ahmed et al. 2023; Bhatt et al. 2024).

**Table 1:** Summary of microplastics detected in the samples anlyzed in this study

Specimens	Collection stations	*n*	DF (%)	# of MPs (items)	Abundance of MPs (items/ind.)		Polymer types *2,3	Shape *2	Size *2
					Mean	Med. (Min.–Max.)			
Freshwater fish (*n* = 72)									
Nile tilapia (*Oreochromis niloticus*)	A, B, C, E, F, G, H, I, J	34	82	349	10 ± 13	4.0 (n.d.−38)	PET (30), PP (28), PE (25)	Fragment (54), Fiber (46)	0.1–0.5 (34), 0.5–1 (27), 1–5 (39)
Adult (body size: > 18.6 cm *4)	B, C, F, G, H, I, J	20	95	319	16 ± 14	12 (n.d.−38)	–	–	–
Juvenile (body size: < 18.6 cm *4)	A, B, E, F	14	64	30	2.1 ± 3.1	1.0 (n.d.−11)	–	–	–
Largemouth bass (*Micropterus salmoides*)	C, D, E, F, G, J	20	40	15	0.76 ± 1.4	n.d. (n.d.−6.0)	PET(46), PE(20), PP,PA,PAN(7)	Fragment(60), Fiber(40)	0.1–0.5 (33), 0.5–1 (33), 1–5 (33)
Snakehead (*Channa argus*)	A, C, D, E, F, G	18	33	8	0.44 ± 0.70	n.d. (n.d.−2.0)	PE(25), PA(13), PAN,PVA(12)	Fragment(62), Fiber(38)	0.1–0.5 (63), 0.5–1 (12), 1–5 (25)
Aquatic grass (*n* = 8)									
Brazilian waterweed (*Egeria densa*)	A	3	100	63	21 ± 12	16 (12–35)	PET (49), PE (24), PAN (10)	Fragment(23), Fiber(77)	0.1–0.5 (14), 0.5–1 (25), 1–5 (60)
Eelgrass (*Vallisneria higoensis*)	F, G	3	100	54	18 ± 12	11 (11–32)	PP(37), PE(24), PET(19)	Fragment(61), Fiber(39)	0.1–0.5 (50), 0.5–1 (30), 1–5 (20)
Holly-leaf waternymph (*Najas marina*)	F, G	2	100	8	4.0	4.0 (4.0–4.0)	PP(100)	Fragment(100)	0.1–0.5(50), 0.5–1 (50)

^*^1: Unit of MP abudance is items/individual for fish specimens and items/g dry weight for aquatic plant samples

^*^2: the values in perenthesis are composition percentage (%)

^*^3: PET: Polyethylene terephthalate, PP: Polypropylene, PE: Polyethylene, PA: Polyamide, PAN: Polyacrylonitrile, PVA: Polyvinyl alcohol

^*^4: The criteria to distingish adult or juvenile was referred to Froese, R. and D. Pauly. Editors. 2024. FishBase. World Wide Web electronic publication. (www.fishbase.org), Nile tilapia (*Oreochromis niloticus*)

Figure 1 illustrates the spatial distribution of MPs in tilapia collected at the different stations in Lake Ezu. High exposure levels were observed at stations G, H, and J (Fig. 1). Station J is located near the outlet of WWTP, which is a large point source of MPs in freshwaters (Talbot and Chang 2022). Dalu et al. (2023) found that the fish inhabiting waters downstream of a WWTP were more highly contaminated with MPs than fish upstream. Accordingly, fish in Lake Ezu near station J are likely impacted by MPs discharged into the receiving water from WWTP. Similarly, station G was located near a stormwater outlet (Fig. 1, SW). The stormwater runoff has been identified as a large non-point source of MPs in aquatic environment (Shafi et al. 2024), with surface runoff estimated as accounting for 99% of the annual MPs emissions of urban catchment into rivers in Japan (Imbulana et al. 2024). The water current in Lake Ezu is from north to south, and hence, MPs inflowing via surface runoff near station G might accumulate near station H, resulting in high level of MPs exposure in tilapia at those sites. This assumed that distribution pattern is consistent with the low level of MPs detected in fish collected at stations E, F, and I.

**Fig. 3:**
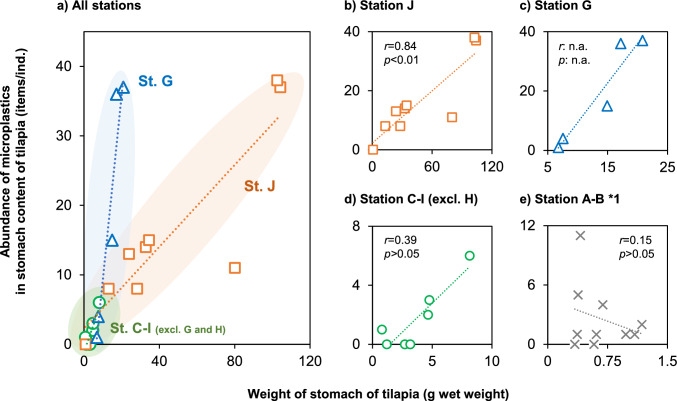
Correlation between MPs abundance in tilapia and weight of stomach in each sampling station. *The statistical values (*r* and *p*) are Spearman’s rank correlation coefficients, n.a.: the statistical values were not available due to the limitation of sample number, *1: removed one outlier (B: 37 items/ind.)

In contrast, the abundance of MPs in largemouth bass and snakehead collected at stations G and J were low, at most 1.0 items ind.^−1^ (Fig. 2; Table S1). If the MP level in the environment samples of surface water was simply associated with the level of MPs in fish, higher levels of MPs would have been detected in largemouth bass and snakehead from station G and J. This indicates that another factor (e.g., dietary habits) can be the cause of differences in the MPs exposure level among different fish species. In addition, some positive correlations for several sampling stations were observed between the MPs abundances in tilapias and the weight of their stomach (namely amount of stomach content) (Fig. 3). These results imply that the exposure pathway to MPs in this species is related mainly to their feeding activity (not water exposure), and that MPs in this fish likely derive from the same source (namely diets) among stations. Lastly, the difference of slope shown in Fig. 3 may also reflect differences of the background contamination levels of MPs in each area of the lake.

### Characteristics of MPs in the Fish

**Fig. 4:**
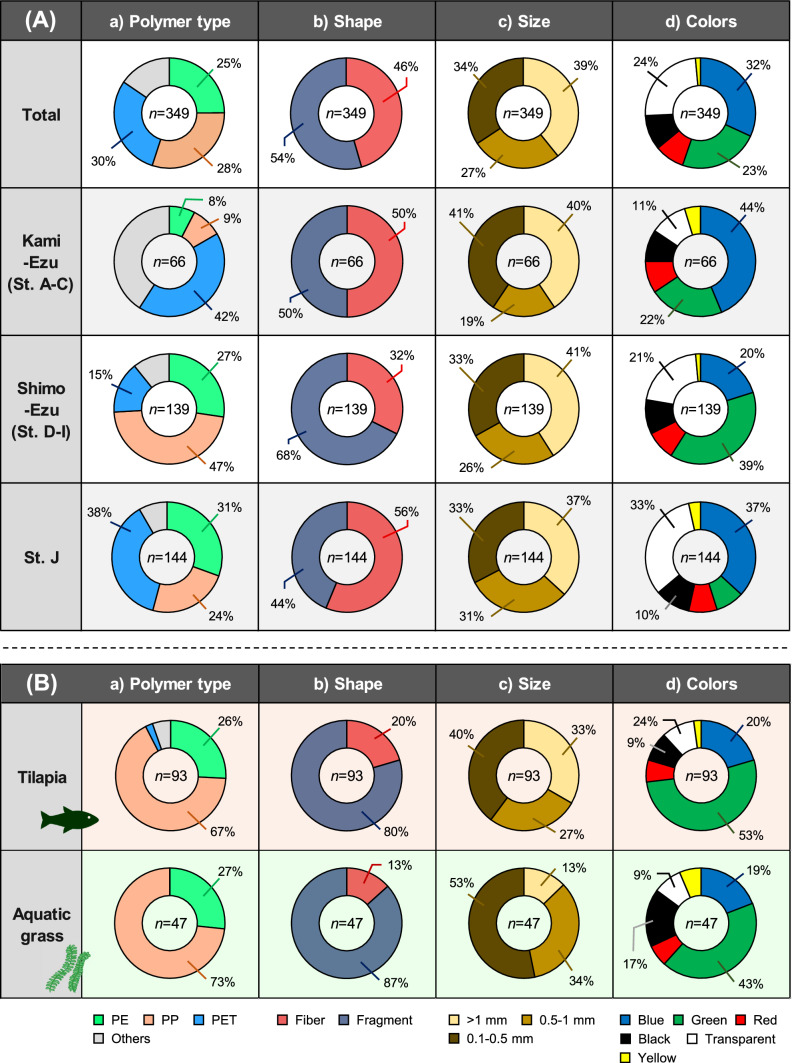
Characteristics of microplastics (a: polymer types, b: shape, c: size, and d: colors). **A** Comparison among tilapias collected from Kami-Ezu (station A–C), Shimo-Ezu (station D–I), and station J. **B** Comparison between tilapias and aquatic plants collected from station G

Ten types of polymers were identified in the fish stomach contents. In tilapia, PET was the most dominant polymer (30%), followed by PP (28%) and PE (25%) (Table 1; Fig. 4A). Although the number of MPs detected was limited in the other two fish species, the polymer profiles were slightly different in largemouth bass (PET: 46%, PE: 20%) and snakehead (PE: 25%, polyamide: 13%, polyacrylonitrile [PAN]: 12%, polyvinyl alcohol [PVA]: 12%) (Table S1). Era and Nakata (2020) investigated MPs in sediments of Lake Ezu and reported a profile of polymers (incl. PE, PP, and PET) similar to that found in the fish analyzed in this study. Nodehi et al. (2024) also noted that those three polymers were frequently found in the fish samples investigated in several previous studies.

MPs with a fragmented shape occupied a slightly larger proportion (54–62%) than fibrous ones (38–46%), and there was no clear difference of MP shapes among the three species of fish (Table 1). The MP size fractions of 0.1–0.5 mm and > 1 mm were dominant, accounting for 67–88% of total MPs (Table 1). Most of all MPs with a size of > 1 mm was fibrous, whereas the other MP sizes often comprised fragments.

By color, blue MPs were the most frequent (31%), followed by transparent/white (24%), green (22%), and black (11%). Okamoto et al. (2022) found that the preferred and most frequently ingested MPs colors by two marine fish species (Indian medaka *Oryzias melastigma* and common clown anemonefish *Amphiprion ocellaris*) were red, yellow, and green. Our results revealed a slightly different color profile of the MPs ingested by the freshwater species, which might imply a degree of unintentional ingestion of MPs by those fish. In addition, color can be used as a potential indicator of inorganic pigments, such as copper phthalocyanine (blue, green), lead chromate (PbCrO_4_; yellow), and titanium oxide (TiO_2_; white) (Yamahara et al. 2024b). A variety of colored MPs identified in fish species therefore implies potential exposure to harmful heavy metals via MPs.

Next, we compared the characteristics of MPs among sampling areas (Kami-Ezu [St. A–C], Shimo-Ezu [St. D–I], St. J) especially in tilapia. The size and color of MPs identified in the fish did not clearly differ among the sampling areas (Fig. 4A). Nonetheless, PET fiber was dominant in Kami-Ezu (58%; 50%) and St. J (38%; 56%), respectively. Textiles are well known as a large source of polyester fibers, including PET (Hernandez et al. 2017). It has been documented that large quantity of MP fibers are produced during washing and drying clothes and are consequently present in effluent discharged from WWTP (Cesa et al. 2017; Iordachescu et al. 2024). Even so, the release of comparable amount of MP fibers from clothes worn in recreational waters should be noted (De Falco et al. 2020). Kami-Ezu is more heavily used by people visiting this area recreationally; hence, clothing worn in the lake might be a potential source of PET in the fish in Kami-Ezu. Similarly, the large proportion of PET fibers found in station J, likely derived from MPs in the WWTP effluent. On the other hand, Shimo-Ezu, mainly station G, showed a different profile of MPs (PP: 47%; fragment: 68%) when compared with Kami-Ezu and station J (Fig. 4A). As mentioned above, a large portion of MPs in Shimo-Ezu are probably derived from stormwater runoff, and thus, the unique profiles obtained from different sources when compared with that in Kami-Ezu at station J.

### Abundance and Characteristics of MPs on Aquatic Plant

A total of 125 MPs were identified in the aquatic plant samples. Brazilian waterweed showed the highest abundance of MPs (mean: 21 ± 12 items g^−1^ dw; med.: 16 items g^−1^ dw), followed by eelgrass (mean: 18 ± 12 items g^−1^ dw.; med.: 11 items g^−1^ dw) and holly-leaf waternymph (med.: 4 items g^−1^ dw) (Table 1). Huang et al. (2023) reviewed published information on the enrichment of MPs in aquatic plants and macroalgae, wherein field studies reported average MPs abundances varying from 0.03 to 33 items g^−1^ dw, suggesting the trapping ability of MPs. The ability of aquatic plant is associated with their structure and specific surface area (Huang et al. 2023). Ng et al. (2022) reported that the filamentous algae could retain 2.35 times more MPs than non-filamentous types, a trend likewise recognized in several other studies (Huang et al. 2023). The aquatic plant analyzed in this study is all species with filamentous structure, allowing a high level of MPs to be retained (Fig. S2). Cozzolino et al. (2020) highlighted that aquatic plant acts as a sink of MPs accumulation in water bodies. Several studies have mentioned that sediment or surface water samples collected from vegetated area have higher abundances of MPs than those of unvegetated area (1.1–5.7 times higher) (Huang et al. 2023; Ng et al. 2022; Cozzolino et al. 2020; Jones et al. 2020). Moreover, approximately 88% of the studies reviewed in Huang et al. (2023) showed higher levels of MPs in aquatic plant samples than in abiotic samples, such as sediment, if the same scale unit (items g^−1^ dw) is applied. Calculating the ratio of MPs in plant/sediment in this study by referring to the sediment data of Era and Nakata (2020) (1.0 items g^−1^ dw for St. A; 0.87 items/g dw for St. F and G) resulted in 12–34 for *Egeria densa*, 13–37 for *Vallisneria higoensis*, and 4.6 for *Najas marina*, which is comparable or apparently greater than the ratios reported in previous studies (Ng et al. 2022; Cozzolino et al. 2020; Battisti et al. 2020; Saley et al. 2019). Although it is problematic to simply compare the concentrations of MPs between the different sample matrices, this result does suggest that aquatic plant acts as a large sink of MPs in the freshwater ecosystems.

Of the total MPs in the aquatic plant samples, PET accounted for 33%, followed by PP (25%) and PE (23%). The polymer profiles (PET, PP, and PE) identified from the aquatic plant samples were consistent with those of sediments (Era and Nakata 2020) and fish analyzed in this study (Table 1). Several studies have revealed a presence of abundant fibrous MPs (71–100%) on aquatic plant (Huang et al. 2023; Cozzolino et al. 2020; Pietrelli et al. 2017; Goss et al. 2018; Seng et al. 2020; Feng et al. 2020; Esiukova et al. 2021; Li et al. 2022), but our results are not necessarily consistent with that trend (Fig. 4B). We speculate that differences in the sampling location might influence the shape profiles of MPs in the aquatic plant samples, the proportion of MPs fiber in the plant samples was 77% in station A versus 33% at station F and G.

### Relationship of MP Profiles between Freshwater Fish and Aquatic Plant

We hypothesized that the feeding of aquatic plant is a key pathway to MPs exposure in herbivorous freshwater fish. A portion of the tilapia and aquatic plant samples were collected at the same time and same points (station G), and Fig. 4B illustrates the characteristics of MPs found in tilapia and aquatic plant collected there. The MPs profiles were quite similar in terms of polymer types, shapes, and colors between tilapia and aquatic plant samples. This suggests that herbivorous tilapia is unintentionally exposed to MPs adsorbed on the aquatic plant through their feeding. Moreover, this is not incompatible with the overall results, indicating that herbivory is strongly associated with exposure to MPs and that the source of MPs in tilapia in Lake Ezu is disproportionately attributed to their food source (Fig. 4).

Most of previous studies have focused on either on the adverse effects of MPs on aquatic plants, the function of vegetated area as a sink of MPs, or differences in MP trapping ability by plant morphology. In addition to those considerations, our study highlights that aquatic plant can facilitate MP pollution in herbivorous freshwater fishes via MPs adhering to the plant sources.

## Supplementary Information

Below is the link to the electronic supplementary material.

Supplementary file1 (PDF 543 KB)
