# Supplementary material for: Aquatic Plant Mediates Microplastic Bioavailability in Herbivorous Freshwater Fish
Source: Arch Environ Contam Toxicol. 2025 Oct 29;89(4):409–19. doi: 10.1007/s00244-025-01164-3 (PMC12665637; doi:10.1007/s00244-025-01164-3)
Supplement: Supplementary file 1 — Supplementary file1 (PDF 543 KB) [file 244_2025_1164_MOESM1_ESM.pdf]

Supplementary materials

Aquatic plant mediates microplastic bioavailability in herbivorous freshwater fish

Shinnosuke Yamahara[a)], Yoichi Era[a)], Haruhiko Nakata[b)]*

a) Graduate School of Science and Technology, Kumamoto University, 2-39-1 Kurokami, Chuo-ku, Kumamoto 860-8555, Japan
b) Faculty of Advanced Science and Technology, Kumamoto University, 2-39-1 Kurokami, Chuo-Ku, Kumamoto 860-8555, Japan

*: Corresponding author (E-mail: nakatah@kumamoto-u.ac.jp)

Contents

Figure S1. Pictures of freshwater fish and aquatic plant analyzed in this study

Figure S2. Comparison of MPs abundances in aquatic plant samples

Table S1. Information of freshwater fish samples

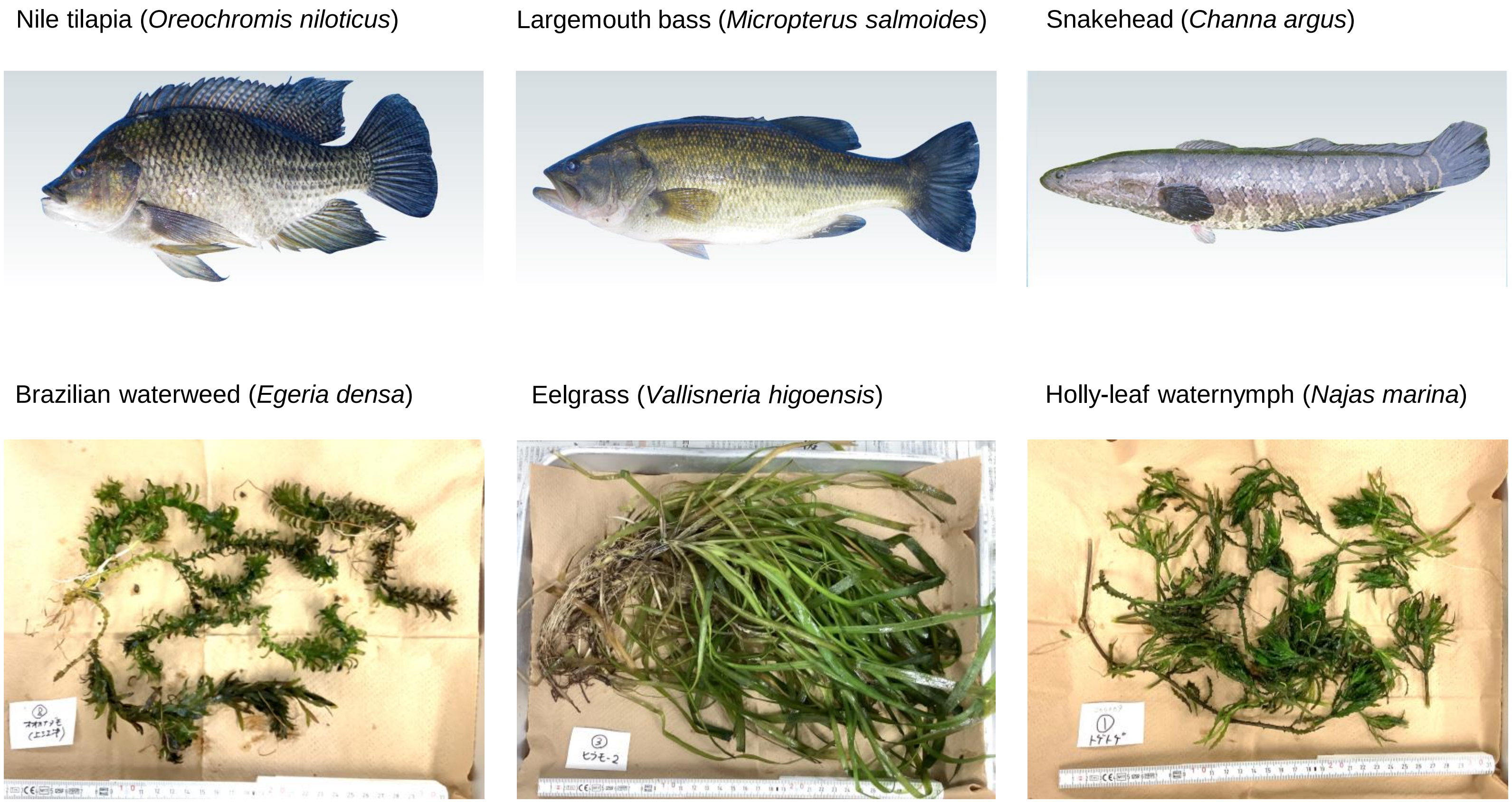

Figure S1. Pictures of freshwater fish and aquatic plant analyzed in this study *The pictures of fish were cited from homepage of Kumamoto City (https://www.city.kumamoto.jp/hpKiji/pub/detail.aspx?c_id=5&id=7881) accessed on 11/11/2024

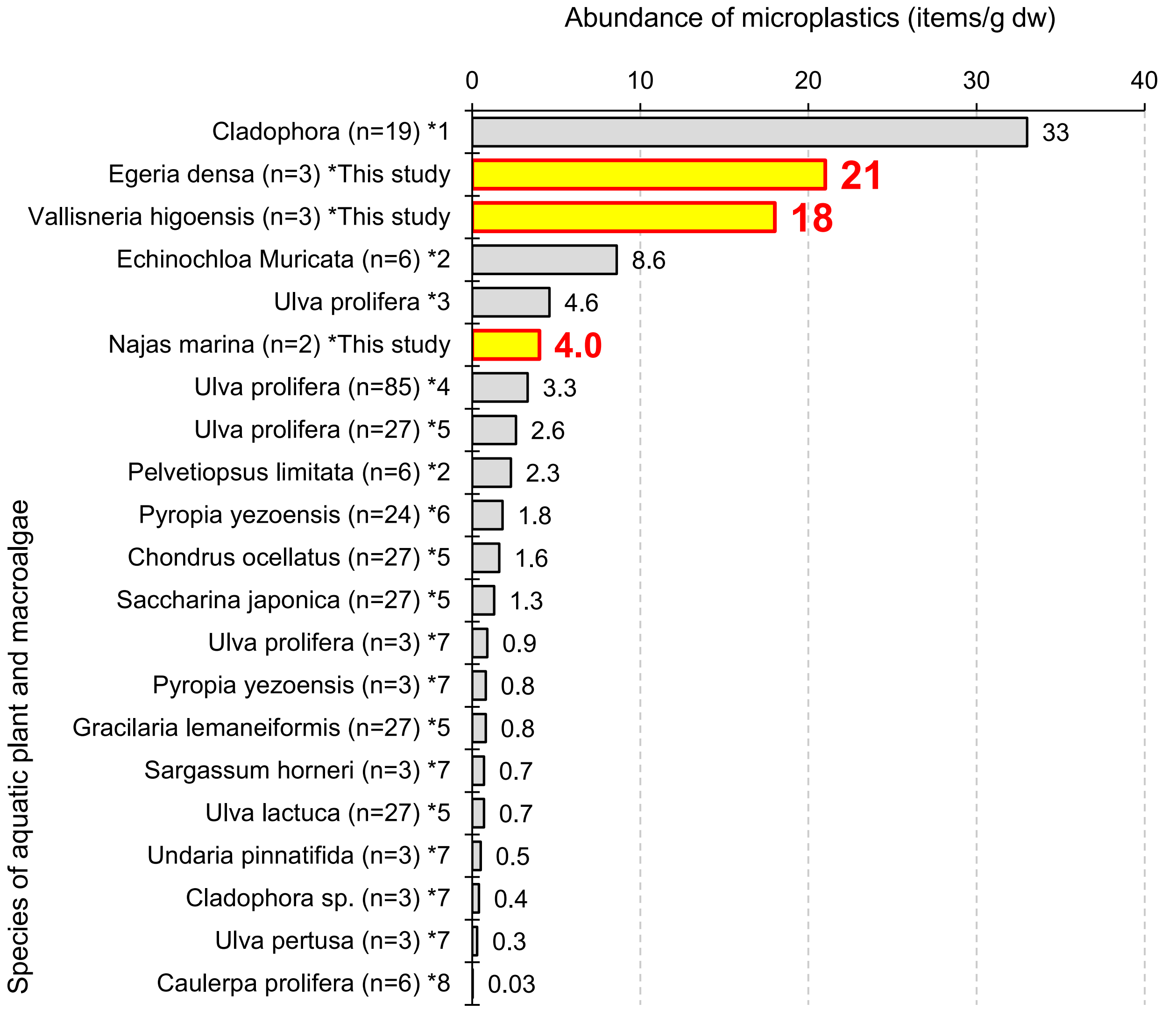

Figure S2. Comparison of MPs abundances in aquatic plant samples. *1: Peller et al., 2021, *2: Saley et al., 2019, *3: Gao et al., 2020, *4: Feng et al., 2020a, *5: Li et al., 2022; *6: Li et al., 2020, *7: Feng et al., 2020b, *8: Cozzolino et al., 2020

Table S1. Information of freshwater fish samples

Sp.	Site ID	Sapling Date (YY/MM/DD)	Whole Size (cm)	Body Size (cm)	Age	Body Weight (g)	Stomach (g wet wt.)	Stomach content (g wet wt.)	Count of MPs (items)	Polymer types				Shape		Size distribution (mm)			Color						
										PE	PP	PET	Other	Fiber	Frag.	>1	0.5-1	0.1-0.5	Blue	Green	Red	Black	White /Trans.	Yellow	Other
**Nile tilapia (*Oreochromis niloticus*; *n* =34)**																									
	A	2020/12/16	11.9	9.8	Juvenile	35	0.41	0.36	11	0	1	6	4	8	3	6	2	3	2	3	1	4	1	0	0
	A	2020/12/23	14.3	11.9	Juvenile	61	0.38	0.11	5	0	2	2	1	2	3	2	1	2	3	1	0	0	1	0	0
	A	2019/10/23	20.8	16.2	Juvenile	158	0.69	n.a.	4	0	0	4	0	4	0	2	2	0	3	0	0	0	1	0	0
	A	2019/11/5	13.4	10.7	Juvenile	41	1.2	n.a.	2	0	0	1	1	0	0	0	0	0	0	0	0	0	0	0	0
	A	2020/12/23	12.4	10.3	Juvenile	45	1.1	0.83	1	0	0	0	1	1	0	1	0	0	1	0	0	0	0	0	0
	A	2020/12/4	11.4	9.6	Juvenile	30	0.61	0.38	1	0	0	0	1	1	0	0	1	0	0	0	1	0	0	0	0
	A	2020/12/16	10.9	8.9	Juvenile	33	0.58	0.44	0	0	0	0	0	0	0	0	0	0	0	0	0	0	0	0	0
	A	2020/12/16	9.4	7.8	Juvenile	18	0.34	0.29	0	0	0	0	0	0	0	0	0	0	0	0	0	0	0	0	0
	B	2020/11/25	9.6	7.9	Juvenile	16	0.37	0.12	1	0	1	0	0	0	1	1	0	0	0	0	0	0	1	0	0
	B	2019/10/23	45.6	37.8	Adult	2,603	54	n.a.	37	5	2	12	18	14	23	12	6	19	17	10	4	2	2	2	0
	B	2019/10/23	42.5	35.4	Adult	1,818	1.0	n.a.	1	0	0	1	0	1	0	1	0	0	1	0	0	0	0	0	0
	C	2020/11/25	33.2	27.0	Adult	821	4.8	1.5	3	0	0	2	1	1	2	1	0	2	1	0	0	0	1	1	0
	E	2020/11/25	14.9	12.1	Juvenile	66	0.79	0.39	1	0	0	0	1	1	0	0	1	0	1	0	0	0	0	0	0
	E	2020/11/25	18.5	15.5	Juvenile	165	3.2	1.9	0	0	0	0	0	0	0	0	0	0	0	0	0	0	0	0	0
	E	2020/12/4	23.5	18.4	Juvenile	325	1.2	0.15	0	0	0	0	0	0	0	0	0	0	0	0	0	0	0	0	0
	F	2020/12/4	35.2	28.5	Adult	1,165	8.1	5.7	6	0	0	3	3	4	2	0	3	3	1	2	1	1	1	0	0
	F	2020/11/25	21.2	17.7	Juvenile	235	2.7	1.3	0	0	0	0	0	0	0	0	0	0	0	0	0	0	0	0	0
	G	2020/7/20	36.8	31.7	Adult	1,036	21	12	37	12	25	0	0	3	34	18	7	12	2	24	2	1	8	0	0
	G	2020/7/20	46.1	38.9	Adult	2,201	17	7.7	36	9	25	0	2	9	27	6	14	16	11	17	4	3	1	0	0
	G	2020/7/20	47.0	38.9	Adult	1,964	15	8.7	15	3	11	0	1	3	12	5	3	7	5	7	0	1	0	2	0
	G	2020/11/25	27.2	22.9	Adult	485	7.5	4.8	4	0	0	2	2	3	1	1	1	2	1	1	0	2	0	0	0
	G	2019/10/23	34.1	28.0	Adult	1,037	6.8	n.a.	1	0	1	0	0	1	0	1	0	0	0	0	0	1	0	0	0
	G	2020/12/4	17.0	13.9	Juvenile	110	1.0	0.57	4	3	0	0	1	4	0	2	1	1	2	0	0	0	2	0	0
	H	2020/7/2	41.1	35.3	Adult	2,463	13	8.9	33	11	3	14	5	16	17	24	6	3	4	3	4	5	17	0	0
	I	2020/11/30	27.5	22.8	Adult	472	4.7	0.73	2	0	0	2	0	1	1	0	0	2	1	0	1	0	0	0	0
	J	2019/11/5	46.9	37.3	Adult	2,068	103	n.a.	38	18	4	14	2	18	20	7	10	21	21	2	4	5	4	0	2
	J	2019/11/5	50.0	42.0	Adult	2,991	104	n.a.	37	15	11	8	3	19	18	21	6	10	11	4	3	0	18	0	1
	J	2020/11/30	46.5	38.4	Adult	2,064	34	23	15	2	5	8	0	11	4	8	6	1	5	1	1	2	6	0	0
	J	2019/11/5	47.0	39.7	Adult	2,083	33	n.a.	14	0	5	8	1	11	3	3	8	3	4	0	1	0	7	0	2
	J	2020/11/30	45.5	38.0	Adult	1,833	24	13	13	3	5	4	1	5	8	7	4	2	2	1	0	3	7	0	0
	J	2019/11/5	49.0	41.0	Adult	3,040	80	n.a.	11	5	0	6	0	6	5	0	5	6	7	2	2	0	0	0	0
	J	2020/10/28	48.9	40.8	Adult	2,926	13	3.8	8	0	2	2	4	5	3	4	1	3	0	0	0	3	5	0	0
	J	2020/10/28	43.7	36.6	Adult	1,859	28	19	8	1	2	4	1	6	2	3	4	1	3	2	1	2	0	0	0
	J	2019/10/16	48.6	40.5	Adult	2,860	0.84	n.a.	0	0	0	0	0	0	0	0	0	0	0	0	0	0	0	0	0
**Largemouth bass (*Micropterus salmoides; n* =20)**																									
	C	2020/11/25	40.6	35.5	n.a.	1,178	53	28	1	1	0	0	0	0	1	1	0	0	0	0	0	0	1	0	0
	C	2020/12/4	39.9	34.8	n.a.	1,102	17	7.3	6	2	1	3	0	3	3	1	2	3	2	1	2	0	1	0	0
	C	2020/11/25	42.2	36.1	n.a.	1,009	17	3.8	1	0	0	0	1	1	0	0	0	1	0	0	0	1	0	0	0
	D	2020/7/2	37.7	33.4	n.a.	809	11	2.1	1	0	0	1	0	1	0	1	0	0	0	0	0	1	0	0	0
	E	2020/11/30	20.9	18.0	n.a.	111	1.5	0.51	0	0	0	0	0	0	0	0	0	0	0	0	0	0	0	0	0
	E	2019/10/23	37.3	31.4	n.a.	766	11	n.a.	3	0	0	3	0	0	3	1	2	0	3	0	0	0	0	0	0
	E	2019/11/5	29.5	24.3	n.a.	348	0.14	n.a.	0	0	0	0	0	0	0	0	0	0	0	0	0	0	0	0	0
	E	2019/11/5	42.5	36.8	n.a.	1,178	0.44	n.a.	0	0	0	0	0	0	0	0	0	0	0	0	0	0	0	0	0

Table S1. Information of freshwater fish samples

Sp.	Site ID	Sapling Date (YY/MM/DD)	Whole Size (cm)	Body Size (cm)	Age	Body Weight (g)	Stomach (g wet wt.)	Stomach content (g wet wt.)	Count of MPs (items)	Polymer types				Shape		Size distribution (mm)			Color						
										PE	PP	PET	Other	Fiber	Frag.	>1	0.5-1	0.1-0.5	Blue	Green	Red	Black	White /Trans.	Yellow	Other
	E	2019/11/5	44.2	37.5	n.a.	1,091	0.92	n.a.	0	0	0	0	0	0	0	0	0	0	0	0	0	0	0	0	0
	E	2019/11/5	44.8	38.3	n.a.	1,344	0.86	n.a.	0	0	0	0	0	0	0	0	0	0	0	0	0	0	0	0	0
	E	2019/11/5	35.0	30.4	n.a.	664	0.56	n.a.	0	0	0	0	0	0	0	0	0	0	0	0	0	0	0	0	0
	F	2020/12/4	24.0	20.5	n.a.	200	1.5	0.10	0	0	0	0	0	0	0	0	0	0	0	0	0	0	0	0	0
	F	2020/11/30	34.0	29.4	n.a.	619	12	6.9	1	0	0	0	1	0	1	0	0	1	0	1	0	0	0	0	0
	F	2020/11/25	34.4	29.9	n.a.	658	9.3	1.5	1	0	0	0	1	1	0	0	1	0	0	0	0	1	0	0	0
	G	2020/7/2	25.1	21.9	n.a.	243	5.1	2.3	0	0	0	0	0	0	0	0	0	0	0	0	0	0	0	0	0
	G	2020/7/2	38.6	34.0	n.a.	834	9.2	1.2	0	0	0	0	0	0	0	0	0	0	0	0	0	0	0	0	0
	J	2019/10/16	28.9	24.5	n.a.	340	1.2	n.a.	0	0	0	0	0	0	0	0	0	0	0	0	0	0	0	0	0
	J	2019/10/23	32.4	27.7	n.a.	484	0.13	n.a.	0	0	0	0	0	0	0	0	0	0	0	0	0	0	0	0	0
	J	2019/10/16	42.0	36.0	n.a.	1,320	0.77	n.a.	1	0	0	0	1	0	1	1	0	0	0	0	0	0	1	0	0
	J	2019/10/16	28.5	24.3	n.a.	315	3.0	n.a.	0	0	0	0	0	0	0	0	0	0	0	0	0	0	0	0	0
Snakehead (*Channa argus*; *n*=18)																									
	A	2019/11/5	66.9	57.7	n.a.	1,865	1.0	n.a.	0	0	0	0	0	0	0	0	0	0	0	0	0	0	0	0	0
	A	2020/12/4	69.6	60.4	n.a.	2,502	32	1.4	0	0	0	0	0	0	0	0	0	0	0	0	0	0	0	0	0
	A	2019/10/16	63.4	55.6	n.a.	1,580	35	n.a.	0	0	0	0	0	0	0	0	0	0	0	0	0	0	0	0	0
	C	2019/10/16	65.9	57.5	n.a.	2,160	2.8	n.a.	0	0	0	0	0	0	0	0	0	0	0	0	0	0	0	0	0
	C	2019/10/23	56.8	48.7	n.a.	1,188	43	n.a.	0	0	0	0	0	0	0	0	0	0	0	0	0	0	0	0	0
	C	2020/12/4	70.0	60.9	n.a.	2,858	46	21	1	0	0	0	1	0	1	1	0	0	0	0	0	0	1	0	0
	C	2020/12/23	56.6	49.6	n.a.	1,516	13	1.0	1	0	0	0	1	1	0	0	1	0	0	0	0	1	0	0	0
	D	2020/7/2	32.5	27.9	n.a.	269	14	4.3	2	1	0	0	1	0	2	0	0	2	0	1	0	0	1	0	0
	E	2020/12/23	64.4	56.1	n.a.	2,477	36	5.4	1	0	0	0	1	1	0	0	0	1	1	0	0	0	0	0	0
	E	2020/12/16	67.5	58.9	n.a.	2,820	67	38	1	0	0	0	1	1	0	1	0	0	0	0	1	0	0	0	0
	E	2019/10/23	59.4	51.8	n.a.	1,557	1.8	n.a.	0	0	0	0	0	0	0	0	0	0	0	0	0	0	0	0	0
	F	2020/11/25	62.1	54.0	n.a.	1,901	19	1.3	0	0	0	0	0	0	0	0	0	0	0	0	0	0	0	0	0
	F	2020/12/4	75.2	65.7	n.a.	3,596	154	110	2	1	0	0	1	0	2	0	0	2	0	0	0	1	1	0	0
	F	2019/11/15	55.0	46.5	n.a.	1,240	1.5	n.a.	0	0	0	0	0	0	0	0	0	0	0	0	0	0	0	0	0
	G	2019/11/15	57.0	48.5	n.a.	1,600	1.4	n.a.	0	0	0	0	0	0	0	0	0	0	0	0	0	0	0	0	0
	G	2019/11/15	89.0	76.0	n.a.	4,070	0.37	n.a.	0	0	0	0	0	0	0	0	0	0	0	0	0	0	0	0	0
	G	2020/10/28	47.7	41.5	n.a.	919	16	4.0	0	0	0	0	0	0	0	0	0	0	0	0	0	0	0	0	0
	G	2020/12/4	58.5	50.1	n.a.	1,146	21	1.7	0	0	0	0	0	0	0	0	0	0	0	0	0	0	0	0	0
